# El Niño Southern Oscillation (ENSO) Enhances CO_2_ Exchange Rates in Freshwater Marsh Ecosystems in the Florida Everglades

**DOI:** 10.1371/journal.pone.0115058

**Published:** 2014-12-18

**Authors:** Sparkle L. Malone, Christina L. Staudhammer, Steven F. Oberbauer, Paulo Olivas, Michael G. Ryan, Jessica L. Schedlbauer, Henry W. Loescher, Gregory Starr

**Affiliations:** 1 Department of Biological Sciences, University of Alabama, Tuscaloosa, AL, United States of America; 2 Rocky Mountain Research Station, US Forest Service, Ft. Collins, CO, United States of America; 3 Department of Biological Sciences, Florida International University, Miami, FL, United States of America; 4 Natural Resource Ecology Laboratory, Colorado State University, Fort Collins, CO, United States of America; 5 Department of Biology, West Chester University, West Chester, PA, United States of America; 6 National Ecological Observatory Network Inc., Boulder, CO, 80301, United States of America; 7 Institute of Arctic and Alpine Research, University of Colorado, Boulder, CO, United States of America; DOE Pacific Northwest National Laboratory, United States of America

## Abstract

This research examines the relationships between El Niño Southern Oscillation (ENSO), water level, precipitation patterns and carbon dioxide (CO_2_) exchange rates in the freshwater wetland ecosystems of the Florida Everglades. Data was obtained over a 5-year study period (2009–2013) from two freshwater marsh sites located in Everglades National Park that differ in hydrology. At the short-hydroperiod site (Taylor Slough; TS) and the long-hydroperiod site (Shark River Slough; SRS) fluctuations in precipitation patterns occurred with changes in ENSO phase, suggesting that extreme ENSO phases alter Everglades hydrology which is known to have a substantial influence on ecosystem carbon dynamics. Variations in both ENSO phase and annual net CO_2_ exchange rates co-occurred with changes in wet and dry season length and intensity. Combined with site-specific seasonality in CO_2_ exchanges rates, El Niño and La Niña phases magnified season intensity and CO_2_ exchange rates at both sites. At TS, net CO_2_ uptake rates were higher in the dry season, whereas SRS had greater rates of carbon sequestration during the wet season. As La Niña phases were concurrent with drought years and extended dry seasons, TS became a greater sink for CO_2_ on an annual basis (−11 to −110 g CO_2_ m^−2^ yr^−1^) compared to El Niño and neutral years (−5 to −43.5 g CO_2_ m^−2^ yr^−1^). SRS was a small source for CO_2_ annually (1.81 to 80 g CO_2_ m^−2^ yr^−1^) except in one exceptionally wet year that was associated with an El Niño phase (−16 g CO_2_ m^−2^ yr^−1^). Considering that future climate predictions suggest a higher frequency and intensity in El Niño and La Niña phases, these results indicate that changes in extreme ENSO phases will significantly alter CO_2_ dynamics in the Florida Everglades.

## Introduction

Teleconnections from the El Niño Southern Oscillation (ENSO) are known to strongly affect climate patterns across North America [1], [2], [3], [4]. ENSO cycles are alternating periods of warm (El Niño phase) and cold (La Niña phase) Pacific Ocean surface temperatures [5], [6], and have occurred with regular periodicity (3 to 7 years) over the last 130,000 years [3]. Shown to influence worldwide precipitation patterns [3], ENSO phases are also correlated with global terrestrial productivity [7] and climate anomalies [3], [8].

In the Florida Everglades, changes in the long-term hydrologic cycle have been linked to extreme ENSO phases (El Niño and La Niña phases) [2], [9]. Precipitation patterns in this region form wet and dry seasons, the frequency and magnitude of which fluctuate with changing climate patterns [8]. Here, El Niño phases increase dry season rainfall causing higher seasonal and annual water levels [2], [9]. In contrast, La Niña phases reduce dry season rainfall, leading to extreme drought and the water table dropping below the soils surface [2], [3], [4], [9]. Because annual shifts in carbon dioxide (CO_2_) exchange rates have been linked to changes in surface hydrology in short-term studies [10], [11], [12], [13], El Niño and La Niña phases may be an important driver of seasonal-to-interannual variations in hydrology and ultimately the productivity of Everglades freshwater marsh ecosystems. It is well known that wetland ecosystem structure and function is tightly coupled to hydrology, and as such it controls wetland carbon (C) sequestration [8], [13], [14], [15]. Wetland CO_2_ exchange rates respond to changes in surface hydrology [11], [13], [16]. The magnitudes of intra- and inter-annual fluctuations in surface hydrology are sensitive to global climate cycles [2], and directly affect CO_2_ exchange. As a result, inter- and intra-annual fluctuations in CO_2_ exchange rates in the Everglades region may be significantly influenced by El Niño and La Niña phases.

Increased atmospheric concentrations of CO_2_ and other greenhouse gases are expected to alter the frequency of El Niño and La Niña phases [17]. In addition to the El Niño and La Niña-driven effects, climate change projections also suggest changes in the magnitude and frequency of seasonal precipitation patterns, as well as higher dry season temperatures [9], [18]. Precipitation projections suggest wetter summers (wet season) and more severe drought (dry season) over the southeastern U.S. [19]. Fluctuations in water availability as a result of these changes may alter ecosystem structure and function.

Surface hydrology is managed differently among watersheds within Everglades National Park, which provides a unique opportunity to examine ecosystem function with differing hydroperiods, while still experiencing similar climate. Schedlbauer et al. [12] and Jimenez et al. [11] have assessed the effects of managed hydroperiods on seasonal and annual carbon (C) dynamics for short periods (1 to 2 years) in Everglades freshwater marsh ecosystems. However, there has been no research to date that has assessed the effects of ENSO teleconnections on seasonal and annual CO_2_ dynamics in the Everglades. Because El Niño and La Niña phases are expected to alter the frequency and intensity of precipitation and temperature regimes, it is unknown how, when, and with what magnitude ecosystem CO_2_ exchange rates will respond to these fluctuations. However, this information is key to develop a prognostic understanding of how these ecosystems will behave in the future.

*The goal of this research is to understand the relationship between extreme ENSO phases and intra- and inter-annual fluctuations in CO_2_ exchange rates (NEE, R_eco_, and GEE).* We hypothesize that El Niño and La Niña will amplify the site-specific seasonal responses in CO_2_ fluxes. At the short-hydroperiod site (Taylor Slough; TS) it has been shown that enhanced net carbon uptake rates are associated with the dry season, while at the long-hydroperiod site (Shark River Slough; SRS) greater net carbon uptake rates are associated with wet season conditions [11], [16]. As El Niño and La Niña phases increase wet and dry season intensity in the Everglades region [2], [3], [4], [9], we expect the site-specific seasonal response to change correspondingly with changes in season intensity. Season intensity here refers to deviations from the mean water availability, so that larger absolute numbers indicate the season intensity and the sign indicates wetter (+) or dryer (-) conditions. We also hypothesize that the variation in season length will explain differences in the interannual CO_2_ dynamics in the respective processes of uptake and efflux. For example, longer and wetter wet seasons will increase the capacity to uptake carbon at long hydroperiod sites, whereas longer and hotter dry seasons will increase the capacity for carbon uptake at short hydroperiod sites. In this study we used the eddy covariance method to estimate whole ecosystem exchanges in CO_2_, and a combination of linear, non-linear and time series modeling techniques to statistically address these hypotheses.

## Materials and Methods

### Study Site

The Florida Everglades are classified as subtropical wetlands with a year-long growing season and distinct wet and dry seasons that define annual variation [20], [21]. Water enters the Everglades through local precipitation events, which average 1380 mm annually [8], and through regional runoff. Presently, water dynamics are controlled by the South Florida Water Management District, which uses a complex system of canals, levees, and pumping stations [8], [22]. The majority of rainfall (∼70%) occurs during the wet season, which begins in May or early June with convective events and tropical depressions, e.g., thunderstorms and hurricanes [8]. Surface water levels generally increase throughout the wet season, are highest at wet season end in October, and decline to their lowest levels by dry season end in May [3]. During the dry season, the Bermuda High-pressure cell prevents convective clouds from forming thunderstorms, making continental fronts the main source of precipitation [20]. This switch from wet season tropical climate to dry season temperate climate causes distinct changes in the amount of precipitation in the region [20]. Dry season precipitation accounts for ∼30% of annual precipitation [8].

**Figure 1:**
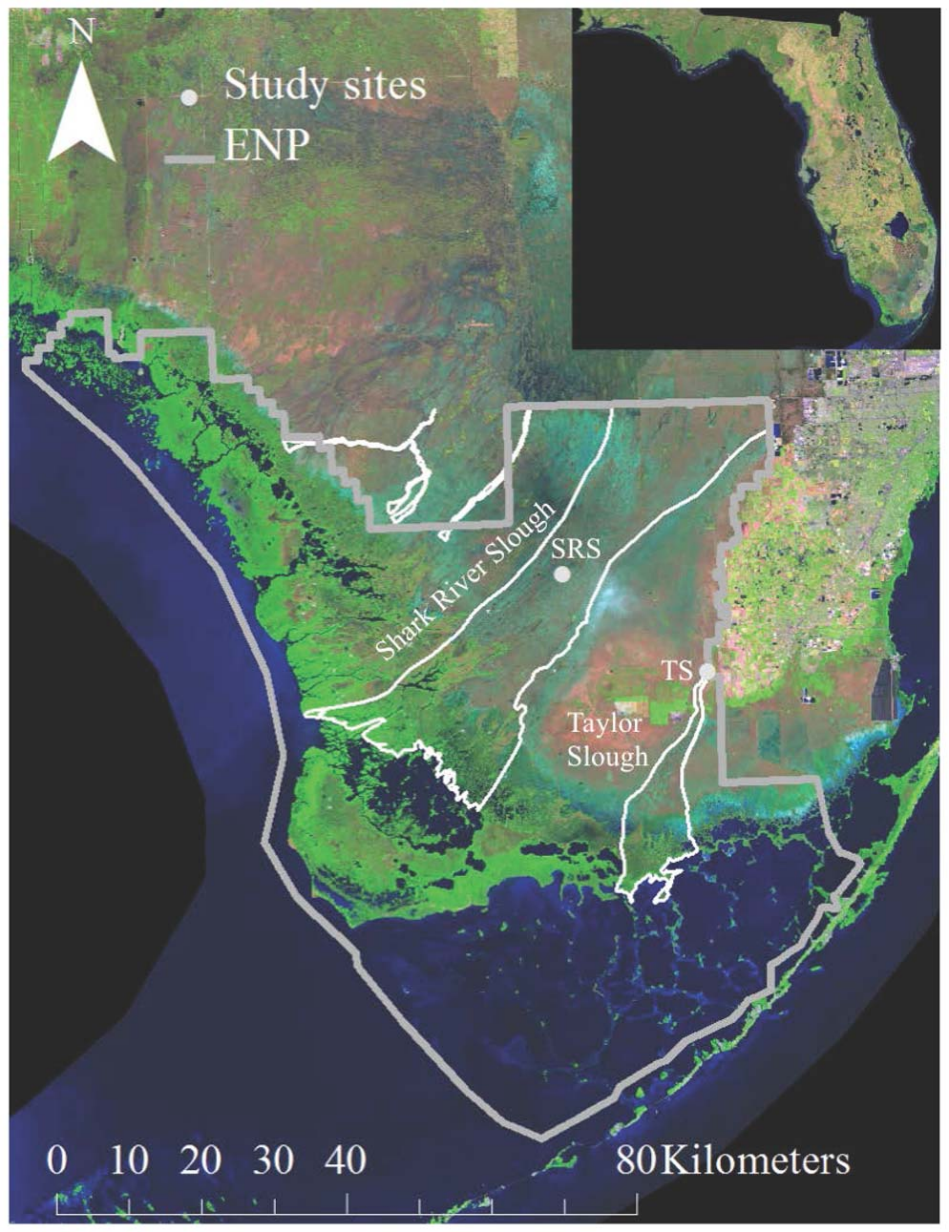
Short- (TS) and long- (SRS) hydroperiod freshwater marsh sites within Everglades National Park, Florida, U.S.A. This map was developed using files created by the Florida Coastal Everglades (http://fcelter.fiu.edu/data/GIS) and LandSat imagery, 2004 LandSat 7 ETM 3-4-5 Statewide Mosaic UTM, made available by the South Florida Water Management District (tp://ftp.sfwmd.gov/pub/gisdata/tm2004_345_mos_utm.zip).

The study sites are two oligotrophic freshwater marsh ecosystems that are within the Florida Coastal Everglades (FCE) long-term ecological research (LTER) program in Everglades National Park (FCE-LTER, http://fcelter.fiu.edu/research/sites/; Fig. 1). Taylor Slough (25°26′16.5″ N, 80°35′40.68″ W) is a short-hydroperiod marsh that is inundated for 4 to 6 months each year (∼June to November) and is characterized by shallow (∼0.14 m) marl soils overlying limestone bedrock. Mean canopy height (Z) and surface roughness (d) for this site are 0.73 and ∼0.3 m, respectively. Shark River Slough (25°33′6.72″N, 80°46′57.36″W) is a long-hydroperiod marsh that is inundated ∼12 months each year and is characterized by peat soils (∼1 m thick) overlying limestone bedrock with ridge and slough microtopography [23]. For this site, Z and d are 1.02 and ∼0.4 m, respectively. Differences in hydroperiod result from spatial variability in elevation [3] and exposure to surface runoff.

In the Florida Everglades, species assemblages and dominance vary with hydrologic patterns [8]. At TS, the continuous homogeneous canopy is dominated by short-statured (0.73 m) emergent species, *Cladium jamaicense* (Crantz) and *Muhlenbergia capillaris* (Lam.) [24], [25]. With ridge and slough microtopography [23] at SRS, tall (1.34 m) dense, emergent species (e.g., *Cladium jamaicense*, *Eleocharis sp*. and *Panicum sp*.) dominate ridge areas. Sloughs are dominated by short-statured (0.70 m), submerged species (e.g., *Utricularia sp*.). Periphyton exists at both sites on submerged structures and as floating mats at SRS (for a more detailed description of the vegetation, see [8]). For this study, data from January 2009 to December 2013 was used and all research was performed under permits issued by Everglades National Park (EVER-2009-SCI-0070 and EVER-2013-SCI-0058).

### Eddy Covariance and Meteorological Data

At each site, open-path infrared gas analyzers (IRGA, LI-7500, Li-COR Inc., Lincoln, NE) were used to measure CO_2_ (c; mg mol^-1^) and water vapor molar density (ρ_v_; mg mol^-1^), and a paired sonic anemometer (CSAT3, Campbell Scientific Inc., Logan, UT) was employed to measure sonic temperature (T_s_; K) and 3-dimensional wind speed (u, v and w, respectively; m s^-1^). These paired sensors were 0.09 m apart and installed at 3.30 and 3.24 m above ground level (a.g.l.) at TS and SRS, respectively. Data were logged at 10 Hz on a datalogger (CR1000, Campbell Scientific Inc.) and stored on 2 GB CompactFlash cards. Both IRGAs were calibrated monthly using a trace gas standard for CO_2_ in air (+1.0%), dry N_2_ gas and a portable dewpoint generator (LI-610, LI-COR Inc.). Footprint analyses [26], [27] indicated that 80% of measured fluxes were within 100 m of the tower during convective conditions at both sites. Other meteorological variables were measured at 1-sec and collected as half-hourly averages, acquired by the same datalogger, and included: air temperature, (T_air_; °C) and relative humidity (Rh; %) (HMP45C, Vaisala, Helsinki, Finland) mounted within an aspirated shield (43502, R.M. Young Co., Traverse City, MI), and barometric pressure (P; atm) (PTB110, Vaisala). The T_air_/Rh sensors were installed at the same height a.g.l. as the IRGA and CSAT.

At each site, additional meteorological data was measured at 15-sec, and collected as 30-min averages through a multiplexer (AM16/32A Campbell Scientific Inc.) with another datalogger (CR10X Campbell Scientific Inc.). This included photosynthetically active radiation (PAR; µmol m^−2^ s^−1^) (PAR Lite, Kipp and Zonen Inc., Delft, Netherlands), incident solar radiation (R_s_; W m^−2^) (LI-200SZ, LI-COR Inc.), and net radiation (R_n_; W m^−2^) (CNR2-L, Kipp and Zonen). Precipitation measurements were made with tipping bucket rain gages (mm) (TE525, Texas Electronics Inc., Dallas, TX). Soil volumetric water content (VWC; %) was calculated from equations developed for peat and marl soils using the methodology of Veldkamp & O'Brien [28], from the dielectric constant using two soil moisture sensors (CS616, Campbell Scientific Inc.) installed at a 45° angle at the soil surface, at each site. Soil temperature (T_s_; °C) was measured at 5 cm, 10 cm, and 20 cm depths at two locations within each site using insulated thermocouples (Type-T, Omega Engineering Inc., Stamford, CT). When inundated at SRS, water temperature, (T_w_; °C) was measured using two pairs of insulated thermocouples (Type-T, Omega Engineering Inc.), each pair located at a fixed height 5 cm above the soil surface and another attached to shielded floats that held the thermocouples 5 cm below the water surface. At TS, T_w_ was measured using insulated thermocouples (Type-T, Omega Engineering Inc.) located at a fixed height 2 cm below the water surface. Water level (m) at both sites was recorded every half-hour with a water level logger (HOBO U20-001-01, Onset, Bourne, MA).

### Data processing

Net ecosystem exchange (NEE) of CO_2_ was estimated through simplification of the continuity equation by applying a control volume approach from the ground level to the top measurement height (*z*; m) [29]. Vertical windspeed (*w*) was first estimated mean to streamline using a 2-d rotation in a Cartesian coordinate framework [29]. NEE (µmol m^−2^ s^−1^) was then estimated using the covariance of the turbulent fluctuations of the vertical rate of change of mean molar density of CO_2_ (*c*′), and the vertical scalar flux divergence (*w*′), where the turbulent fluctuations are the instantaneous deviation (at 10 Hz) from the mean block average (term I) over 30 min, and the storage flux (term II):
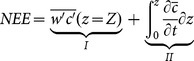
(1)where,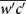 is the measured covariance (m s^−1^ µmol C mol^−1^) of the molar density of CO_2_ measured at a fixed plane above the plant canopy (Z), z is the vertical dimension, and the overbar is the averaging period, in this case is 30-min. NEE was then divided by the molar volume of air, V, (m^3^ mol^−1^) to convert the units from density to molar fraction, i.e., µmol CO_2_ m^−2^ s^−1^, such that:
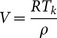
(2)where, *R* is the ideal gas constant (0.082 L atm K^−1^ mol^−1^), *P* is atmospheric pressure (1.10325 atm), and *T_k_* is the actual air temperature, estimated by:
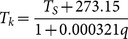
(3)where, *q* is the molar fraction of water vapor calculated by unit conversion of *ρ_v_*. Micrometeorological convention is used here, where negative NEE values indicate ecosystem uptake of CO_2_.

Sensible heat (*H*; W m^−2^) was determined from the covariance of the turbulent fluctuations of *w* and *T_s_*, and *w* and *q* (noted as primes, rf. [29]) estimated over a 30-min averaging period (noted as overbar), such that, 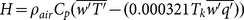
(4)where, *ρ_air_* is the air density (kg m^−3^) and *C_p_* is the specific heat of air at constant pressure (J kg^−1^ °C^−1^). Corrections for the effect of water vapor on the speed of sound were applied [30].

Similarly, latent energy (*LE*; W m^−2^) was calculated from the covariance of the turbulent fluctuations of *w* and *ρ_v_* and averaged over 30-min,
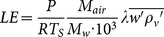
(5)where, *λ* is the heat of vaporization (J g^−1^), and *M_air_* and *M_w_* are the molecular weights of air (28.965 g mol^−1^) and water (18.01 g mol^−1^), respectively. Corrections for thermal and pressure related expansion and/or contraction, and water dilution were applied [31].

10 Hz raw flux data were processed with EdiRe (v. 1.4.3.1184, [32]), which included despiking and air density corrections [31], [33]. Fluxes (NEE, H, LE) were then corrected for mass transfer resulting from changes in density not accounted for by the IRGA [31], [34], and barometric pressure data were used to correct the fluxes to standard atmospheric pressure. All measurements were filtered when systematic errors in either NEE, H or LE were indicated, such as: (1) evidence of rainfall, condensation, or bird fouling in the sampling path of the IRGA or sonic anemometer, (2) incomplete half-hour datasets during system calibration or maintenance, (3) poor coupling of the canopy with the external atmospheric conditions, as defined by the friction velocity, u*, using a threshold <0.15 m s^−1^ [35], [36], and (4) excessive variation from the half-hourly mean based on an analysis of standard deviations for *u*, *v*, and *w* wind and CO_2_ statistics. Quality assurance of the flux data was also maintained by examining plausibility tests for implausible H (<−100 or >800 Wm^−2^), LE (<−100 or >800 Wm^−2^), and NEE (i.e., NEE <−30 or >30 µmol m^−2^ s^−1^) values, stationarity criteria, and integral turbulent statistics [37], [38]. At TS, 38% and 77% of the day and nighttime data were removed, respectively. At SRS, 34% of daytime data and 70% of nighttime data were removed. Missing H and LE were then gap-filled using the linear relationship between H or LE and R_n_ on a monthly basis. When R^2^ values were less than 70%, annual relationships between R_n_ and H or LE were used to gap fill data in that month.

Missing half hourly NEE data were gap-filled using separate functions for day and night. When PAR was ≥10 W m^−2^, NEE data was gap-filled using a Michaelis-Menton approach (*NEE_day_*; Eq. 6), and when PAR was <10 W m^-2^, NEE data was gap-filled using an Arrhenius approach (*NEE_night_*; Eq. 7):
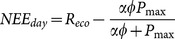
(6)where, *α* is the apparent quantum efficiency (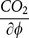), *φ* is *PAR*, *R_eco_* is ecosystem respiration (µmol CO_2_ m^−2^ s^−1^), and *P_max_* is the maximum ecosystem CO_2_ uptake rate (µmol CO_2_ m^−2^ s^−1^).
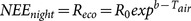
(7)where, *R_0_* is the base respiration rate when air temperature is 0°C and *b* is an empirical coefficient. In equation 6, *R_eco_* is an estimated model parameter, whereas *R_eco_* measurements are the dependent variable in equation 7. A bootstrap method was used for error estimation of gap-filled values of NEE. Synthetic datasets (1000) of size *n* (with replacement) from the original dataset of size *n* for each estimated gap-filling model (Eq.6 and Eq.7) on a monthly or annual basis where appropriate were used [11]. Distributions of each model parameter were constructed, which were then checked to ensure that the model parameters derived from the original data were contained within a 95% confidence region. Following gap filling, GEE was calculated from half hourly *NEE* and *R_eco_* data (Eq. 8).
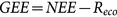
(8)Gap-filled flux data for TS and SRS are made available through AmeriFlux (http://ameriflux.ornl.gov).

### Defining Seasons

**Figure 2:**
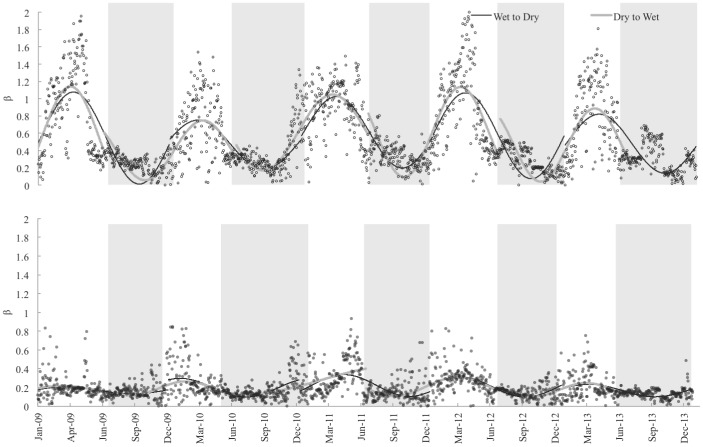
Time series of β for (a) TS and (b) SRS. β was used to determine the change from dry to wet, and wet to dry seasons. Sine functions were fit to the β time series by year and by sites. The initiation of the dry season was determined by fitting a sine function annually (Jan 1 to Dec 31). The inflection point along the positively sloped portion of each sine function identified the change from dry to wet season. The initiation of the wet season was defined by fitting a sine function offset by −182 days (∼6 months), and determining the inflection point along the negatively sloped portion of each sine function. The shaded region highlights the wet season.

Although the majority of rain in the Everglades region falls in the wet season, it is difficult to identify the exact onset of the wet season. Previous studies define season based on the calendar year [39] or water levels [11], [12]; however, these approaches either do not capture interannual variations or are heavily influenced by water management activities performed by the South Florida Water Management District. To determine the date of the shift in seasons we examined the seasonal pattern of Bowen ratios over time,
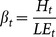
(9)where, the subscript *t* denotes the *t* ^th^ daily value in the time series. Similar to methods used by Nuttle [40] to define hydroperiods, a harmonic function (sine function) was fit to the β time series to identify inflection points that indicate changes in the seasonal trend of the ratio of energy dissipation as *H* and *LE* (Fig. 2). A sine function was fit to the β time series at each site annually (Jan 1 to Dec 31), and the inflection point along the positively sloped portion of each sine function was used to identify the change from dry to wet season (Fig. 2). The wet season was defined by fitting the sine function to the same set of site-specific series offset by −182 days (∼6-months) and identifying the inflection point along the negatively sloped portion of each sine function. The sine function was offset by −182 days so that the shifts in season would not occur near the end of the time series. Previous studies show marked seasonal shifts in energy dissipation in short and long hydroperiod marsh ecosystems (S. Malone, unpublished data) and therefore this method of seasonal classification should adequately capture the seasonality in both water and energy availability.

**Figure 3:**
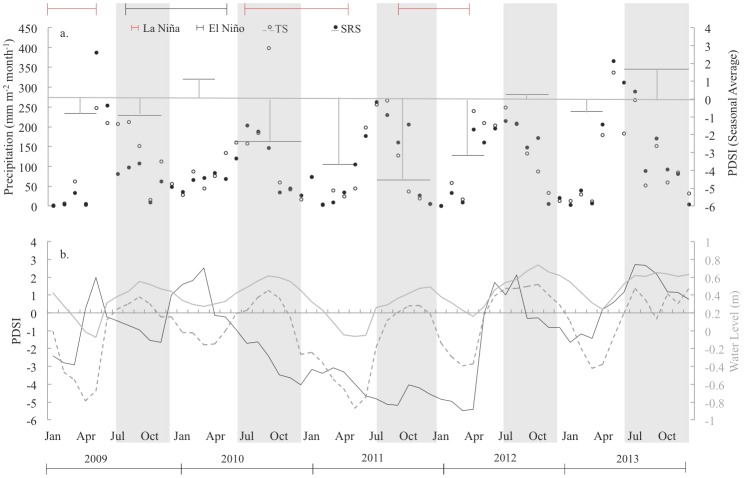
Time series of precipitation, PDSI, and water levels at TS and SRS. (a) Shows monthly cumulative precipitation (mm) and season intensity (as measured by seasonal average PDSI), (b) shows monthly average Palmer Drought Severity Index (PDSI), water level (m), and El Niño and La Niña phase.

As an indication of season intensity, the seasonal mean Palmer Drought Severity Index was used (PDSI; Fig. 3a) [41]. PDSI compares weather conditions to historical weather data, taking into account temperature, rainfall, and the local available water content of the soil. PDSI uses 0 to identify normal conditions, negative numbers (−1 to −6) to indicate dryer than average conditions, and positive numbers to reflect excess rain (Fig. 3). PDSI data were retrieved from the National Climatic Data Center (http://www.ncdc.noaa.gov/temp-and-precip/drought/historical-palmers.php).

### Long-term weather data

Long-term weather data were obtained from the nearest weather station, NCDC Royal Palm Ranger Station (25°23′N/80°36′W), where NOAA surface meteorological data was available from 1964 to 2013. The Oceanic Niño Index (ONI) was used to define extreme ENSO phases and was retrieved from the National Oceanic and Atmosphere Administrations Earth Physical System Research Laboratory (http://www.esrl.noaa.gov/psd/data/climateindices). The ONI is the running 3-month mean sea surface temperature (SST) anomaly from a 30-year mean SST for the Niño 3.4 region (i.e., 5°N-5°S, 120°-170°W). Cold (warm) phases are defined as 5 consecutive months at or below (above) the -0.5° (+0.5°) anomaly.

### Data Analysis

#### Long-term patterns in monthly weather data

An intervention time series approach was used [42], utilizing autoregressive integrated moving average (ARIMA) models and the SAS procedure PROC ARIMA (version 9.3, SAS Institute Inc., Cary, North Carolina) to model 3 variables describing weather for the site, (e.g., monthly precipitation, average maximum daily temperature, and average minimum daily temperature) as a function of ENSO phase. ARIMA models incorporate 3 types of processes: autoregressive (AR) of order *p*, moving average (MA) of order *q*, and if necessary, differencing of degree *d* [42]. ARIMA models fit to time series data use AR and MA terms to describe the serial dependence, and use other time series data from independent variables to explain the dependence on outside factors [42]. The advantage of using ARIMA models is that the internal structure of the data (e.g., autocorrelation, seasonality) is explicitly accounted for by incorporating past values. For example, a first-order autoregressive moving average, or ARIMA(1,1) model, predicts the current time period value (*Y_t_*) using its one period previous value (*Y_t_*_-1_) and its associated error (*ε*_t-1_):
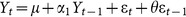
(10)where: μ is the mean of the series, *α*_1_ is the first-order AR coefficient, *θ* is the first-order MA coefficient, *ε*_t_ is the current period error, and *ε*_t-1_ is its one period previous error. “Memory” can be added to the model by adding lags and associated MA and/or AR components, and potential covariates can also be added as predictor variables, which may also have lagged components.

To facilitate the inclusion of independent categorical variables for ENSO, El Niño and La Niña phases were coded as indicator variables, where a value 0 or 1 specified the absence or presence, respectively, of a categorical effect. We then determined if there were teleconnection lags between ENSO phase and precipitation and temperature.

In developing time series models, first, all data series were tested for stationarity via the augmented Dickey-Fuller unit root test [43]. The ARIMA models were then fit to time series data (monthly precipitation, and minimum and maximum temperature) using an iterative Box-Jenkins approach, where: (1) autocorrelation and partial autocorrelation analysis were used to determine if AR and/or MA terms were necessary for the given time series, (2) model coefficients were calculated using maximum likelihood techniques and, (3) autocorrelation plots of model residuals were examined to further determine the structure of the model [42].

Because of the presence of autocorrelation in the explanatory series, input series were pre-whitened [42]. ARIMA models were then fit to the dependent variables using the pre-whitened explanatory series as predictor variables. Cross-correlation coefficient plots between the explanatory series and dependent variables were used to identify direct and inverse relationships at various lags or time shifts, and autocorrelation plots of the residuals verified that the residual series had characteristics of random error, or white noise [42]. Model selection was based on minimum Akaike's information criterion (AIC) and models were acceptable when residual white noise was minimized [44]. A backwards selection method was used, removing the least significant parameter one at a time until all regression terms in the final model were significant at the α = 0.05 level and the lowest AIC was achieved. ARIMA assumptions of normality and independence of residuals [42] were verified by examining residual plots.

#### Seasonal light and temperature response

To examine changes in the response of NEE and R_eco_ to light and temperature, respectively, non-linear equations (6) and (7) were fit. Parameters for these models were fit by ENSO phase, site and season via the SAS procedure PROC NLIN (version 9.3, SAS Institute Inc., Cary, North Carolina). Parameter estimates were then compared to identify differences and similarities. As a result of the high degree of autocorrelation inherent in NEE time series from half-hourly data, the standard errors of parameter estimates from these models are artificially small, and statistical tests are not valid. Therefore, this analysis is presented in a descriptive context.

#### Daily CO_2_ and water dynamics

An intervention time series approach was used to identify and model the relationship between CO_2_ dynamics (*NEE, GEE*, and *R_eco_*) and a set of explanatory variables over a 5-year time series of daily data (2009 to 2013). These variables included: water level, season, ENSO phase (El Niño, La Niña and neutral), daily precipitation, drought condition, and average air temperature. The combined effect of ENSO phase and season (e.g. El Niño × wet season and El Niño × dry season) on CO_2_ fluxes were included in time series models as predictors with indicator variables. Indicator variables were also developed to identify sections of each season that directly followed an ENSO phase (post-La Niña and post-El Niño). Beckage et al. [3] found the effect of extreme ENSO phases during seasonal transitions, and post-La Niña and post-El Niño phases capture the transition periods. To explore the effect of precipitation on CO_2_ exchange rates, indicator variables were used to identify the day of a precipitation event (*Rain Day*), the day after precipitation, and the quantity of precipitation (*Rain*; mm). The indicator for the day after a precipitation event identified the first rain free day following a day with precipitation. Finally, drought conditions were defined as those days where PDSI<-2 and verified the drought extent with the National Oceanic and Atmospheric Administration's Drought Monitor (Fig. 3b; [45]). Drought Monitor data was obtained from the National Drought Monitor Center (http://droughtmonitor.unl.edu/DataArchive.aspx).

In addition, previous studies identified water level as one of the most important drivers for CO_2_ exchange rates in the Everglades freshwater ecosystems [11], [16]. Thus, a water index equal to the difference between each half-hourly water level and its site-specific annual seasonal mean water level was computed. Using the water index as a dependent variable in an additional analysis, time series models were estimated to answer questions about the relationship between intraseasonal fluctuations in water levels, precipitation, and ENSO phase.

As in the models of monthly weather data, all daily time series were tested for stationarity and non-stationary series were made stationary by differencing [46]. ARIMA models were then fit to time series data (*NEE, GEE, R_eco_*, and the *Water Index*) using pre-whitened explanatory series as predictor variables. Cross-correlation coefficient plots identified relationships at various lags, and autocorrelation plots were used to verify that the residuals had characteristics of random error. Model selection was based on minimum AIC, removing the least significant parameters. For each dependent variable, a single model form was selected with common predictor variables to aid site comparisons. Non-significant parameters remained in the model only if the parameter was significant at one site and it did not affect the final model of the other site. Multicollinearity between explanatory variables was also explored to ensure models did not contain input series that were highly correlated.

## Results

### Long-term weather patterns

The long term ONI data ranged from 2.5 to −2.05 and indicated that the neutral phase occurred over about half (46%) of the period 1964–2013, while the El Niño and La Niña phases occurred 26 and 27% of this long-term study period, respectively. Neutral phases ranged in length from ∼1 month to>12 months and just 6 years contained no neutral phase (1969, 1971, 1975, 1987, 1999, and 2000). There were 14 different El Niño and 14 La Niña events, and the average ONI index was ∼1.1, −1, and −0.02 for all El Niño, La Niña and neutral phases, respectively. Although it is common for an El Niño event to be separated from a La Niña event by a short neutral phase (14 occurrences), a neutral phase occurring between consecutive El Niño (1 occurrence) or La Niña phases (4 occurrences) were less frequent. The majority (∼52%) of wet season months (May to October) were associated with a neutral phase while just ∼25% and ∼23% of wet season months occurred during El Niño and La Niña phases, respectively. Dry season months (September to April) were associated with the neutral phase ∼41% of the time, while El Niño and La Niña phases occurred 27 and 32% of time. Time series analysis of long-term monthly precipitation and minimum and maximum daily temperatures versus ENSO phase showed that rain increased the month after the start of El Niño phases (i.e., at a lag of 1 month; p = 0.1043) and declined the month following the start of La Niña (p = 0.7719), though not significantly (Table 1; S1 Fig.). Monthly average maximum daily temperatures were lower the month following the start of La Niña (p = 0.0001), and during El Niño phases (p<0.0001) and at a lag of 1 month (p = 0.001), compared to neutral phases. Average minimum daily temperatures were lower the month following the start of El Niño (p = 0.019) and La Niña (p = 0.0218; Table 1) phases.

**Table 1:** Parameter estimates from ARIMA models of monthly precipitation, and average daily maximum and minimum temperature.

	Precipitation				Temperature (max)				Temperature (min)			
Parameter	Estimate	Standard Error	t Value	Approx	Estimate	Standard Error	t Value	Approx	Estimate	Standard Error	t Value	Approx
				Pr>|t|				Pr>|t|				Pr>|t|
MA(1)	0.2691	0.0515	5.23	<.0001	−0.3868	0.0394	−9.83	<.0001	−0.3018	0.0399	−7.57	<.0001
AR(1)	0.4677	0.0397	11.79	<.0001	0.9972	0.0025	397.31	<.0001	0.9868	0.0064	154.31	<.0001
AR(2)	0.4786	0.0352	13.60	<.0001								
El Niño	−9.2460	16.8264	−0.55	0.5827	−1.3567	0.2735	−4.96	<.0001	−1.7009	0.4508	−3.77	0.0002
El Niño (1)	27.1448	16.7098	1.62	0.1043	−0.8904	0.2714	−3.28	0.001	−1.0517	0.4484	−2.35	0.019
La Niña	13.9136	17.5394	0.79	0.4276	−0.3878	0.2805	−1.38	0.1667	−0.1130	0.4568	−0.25	0.8047
La Niña (1)	−5.05061	17.4237	−0.29	0.7719	−0.9126	0.2809	−3.25	0.0012	−1.0465	0.4564	−2.29	0.0218

MA(1) is the estimated moving average term at a 1- period lags (1 month) and AR(1) and AR(2) are the estimated autoregressive term at a 1- and 2-period lags (1 and 2 months, respectively). Lagged values of independent variables are denoted similarly. *El Niño* is an indicator for El Niño phases and *La Niña* is an indicator for the La Niña phase, determined by the ONI index.

### Shifts in ENSO phase and site conditions (2009–2013)

Throughout the study (2009–2013), 3 short La Niña phases (2009, 2010–2011 and 2011–2012), and an El Niño phase (2009–2010) occurred with shifts in precipitation patterns that resulted in both wetter than average (2010) and drier than average years (2009 and 2011; Fig. 3a). The observed sequence in ENSO phases from 2009 to 2013 has occurred twice (1984–1990; 1997–2001) over the study period. While there has been a cooling trend in the Pacific Ocean since 2007, the El Niño phase in 2009–2010 was short with weak to moderate strength. In 2013, both the wet and dry seasons were in a neutral phase. Neutral phases greater than 12 months were not uncommon over the study period (1964–2013; 9 occurrences). The mean ONI indices during 2009–2013 were similar to that of the 40-year period, with values of 1, −1, and −0.2 for the El Niño, La Niña and neutral phases, respectively. Season intensity, defined by the seasonal average PDSI for the Everglades region, changed with ENSO phases (Fig. 3a). During La Niña phases, seasonal mean PDSI ranged from ∼−1 to −4 (Fig. 3a). In the wet season of 2009 an El Niño phase began soon after a La Niña phase. The El Niño phase extended into the dry season of 2010 where it co-occurred with wetter than average conditions (PDSI>1). During the neutral phase in 2013, mean wet season PDSI was 1.7 and −0.6 in the dry season.

**Figure 4:**
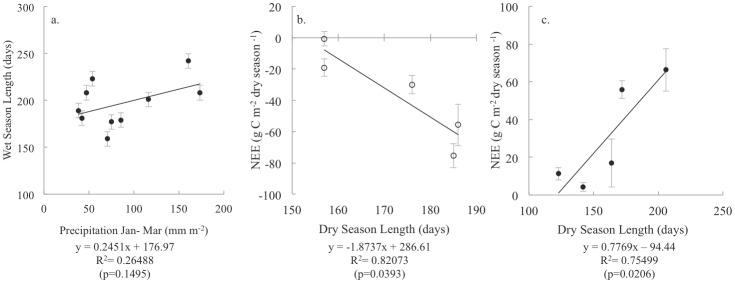
Precipitation, season length, and CO_2_ Exchange rates. (a) The relationship between cumulative precipitation (January through March) and wet season length at TS and SRS shows that precipitation prior to the wet season is an important determinant of wet season length. The relationship between dry season length and dry season NEE is positive at (b) TS and negative at (c) SRS.

Although TS and SRS had similar weather, harmonic analysis of β (Fig. 2) showed that both hydroperiods and season length differed annually and between sites (Fig. 3b). The onset of the wet season at TS lagged SRS by approximately one month on average, with wet season length varying between 179 to 208 days at TS and 159 to 242 days at SRS. Wet season length was positively correlated with cumulative precipitation from January to March (p = 0.1495; Fig. 4a). During abnormally dry years (PDSI<−2), the wet season was shortened by about 15 days at TS and 34 days at SRS compared to all other years. In 2009 and 2011, south Florida experienced severe drought conditions (water levels below the soil surface) resulting in 65 and 34 dry days at TS and SRS, respectively (Figs. 3 and 4). Drought years were not characterized by lower annual precipitation but by lower rainfall and fewer rain events the 3 months prior to the start of the wet season, which generated a shorter season (Fig. 4a). In 2010, 2012 and 2013, total rainfall in the first 4 months of the year averaged 261 mm at TS and 248 mm at SRS, while during drought years (2009 and 2011) TS and SRS received just 107 mm and 82 mm on average, respectively.

### Annual and Seasonal patterns in CO_2_ Fluxes

Annual net CO_2_ exchange rates at both freshwater marsh ecosystems co-varied with ENSO phase (2009–2013), which corresponded to changes in season intensity. The ratio of GEE to R_eco_ suggests that CO_2_ exchange rates at both sites were most similar in 2010 (El Niño; S2 Fig.) when hydraulic conditions were comparable between TS and SRS. The lowest rates of NEE (greatest CO_2_ uptake) occurred during the La Niña phase at TS followed by the neutral phase. At SRS the greatest CO_2_ uptake rates were during the El Niño and neutral phase compared to seasons associated with a La Niña phase (Table 2). The annual ratio of GEE to R_eco_ and annual rates of NEE both show that sites were most similar when water levels were higher and during the neutral phase (S2c Fig.).

**Table 2:** Seasonal and annual NEE, GEE, and R_eco_ (g C m^−2^ yr^−1^) at Taylor Slough and Shark River Slough.

		TS								SRS						
Year	Season	NEE (S.E)		GEE (S.E)		R_eco_ (S.E)		Season Length	ENSO	NEE (S.E)		GEE (S.E)		R_eco_ (S.E)		Season Length
2009	Dry	−30.0	(5.9)	−261.9	(5.8)	231.9	(7.3)	176	*	66.3	(11.3)	−163.0	(11.6)	229.3	(5.2)	206
	Wet	19.0	(7.8)	−194.2	(7.1)	213.2	(8.8)	189	•	13.7	(3.6)	−198.3	(5.4	211.9	(4.5)	159
	Annual	−11.0	(13.7)	−456.1	(12.9)	445.1	(16.1)			80.0	(14.9)	−361.3	(17.0)	441.3	(9.7)	
2010	Dry	−19.1	(5.6)	−199.2	(4.2)	180.1	(5.3)	157	•	−11.3	(3.4)	−249.3	(3.3)	238.0	(1.9)	123
	Wet	13.8	(7.1)	−219.3	(6.0)	233.1	(7.0)	208	*	−4.7	(8.3)	−92.3	(7.4)	87.6	(5.8)	242
	Annual	−5.3	(12.7)	−418.5	(10.2)	413.2	(12.2)			−16.0	(11.6)	−341.6	(10.8)	325.6	(7.7)	
2011	Dry	−55.7	(13.3)	−302.7	(14.0)	246.9	(11.6)	186	*	16.9	(12.7)	−230.9	(7.0)	247.7	(10.8)	164
	Wet	−54.8	(14.8)	−308.6	12.8)	253.8	(15.4)	179	*	59.5	(7.3)	−151.6	(7.4)	211.2	(7.4)	201
	Annual	−110.5	(28.1)	−611.3	(26.8)	500.8	(27.0)			76.4	(20.0)	−382.5	(14.4)	458.9	(18.1)	
2012	Dry	−75.4	(7.6)	−249.9	(7.9)	174.6	(7.7)	185	*	55.9	(4.7)	−115.8	(3.4)	171.7	(3.1)	172
	Wet	31.5	(8.3)	−123.3	(8.3)	154.9	(8.0)	181		8.5	(7.2)	−137.0	(5.0)	145.5	(4.7)	194
	Annual	−43.8	(15.9)	−373.2	(16.2)	329.4	(15.7)			64.5	(11.9)	−252.8	(8.4)	317.3	(7.8)	
	Dry	−0.57	(4.6)	−119.2	(3.5)	118.6	(4.6)	157		−4.3	(2.4)	−97.7	(2.0)	93.4	(2.5)	142
2013	Wet	−30.0	(6.8)	−146.9	(4.6)	116.8	(5.8)	208		6.1	(5.1)	−137.9	(4.1)	144.0	(4.3)	223
	Annual	−30.6	(11.4)	−266.1	(8.1)	235.5	(10.4)			1.8	(7.5)	−235.6	(6.1)	237.5	(6.8)	

Seasons with a La Niña or El Niño phase are marked with an * and •, respectively.

**Figure 5:**
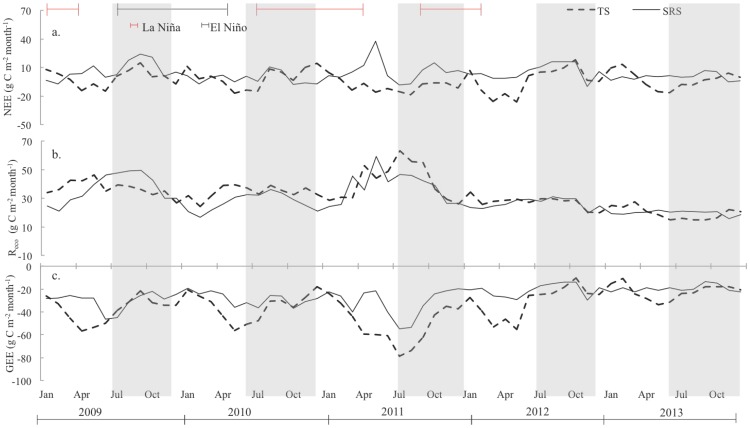
Time series of monthly (a) NEE, (b) R_eco_ and (c) GEE for TS and SRS. Variations in both ENSO phase and CO_2_ exchange rates co-occurred with changes in wet and dry season length.

**Figure 6:**
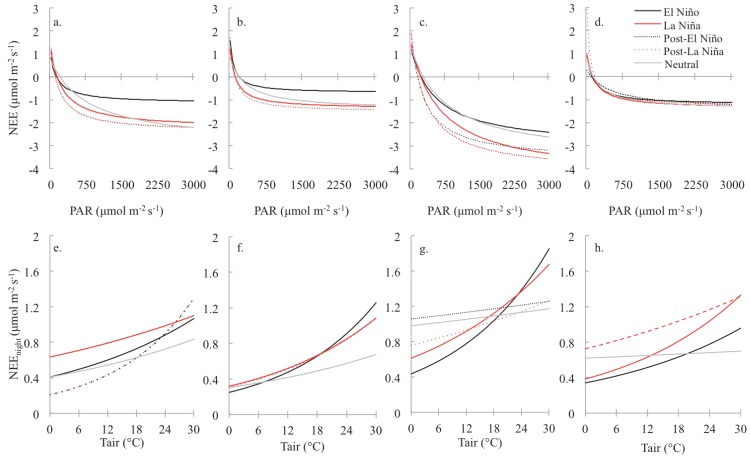
Light and Temperature Response Curves. Light response curves showing differences in photosynthetic capacity by ENSO phase during the wet season at (a) TS and (b) SRS, and during the dry season at (c) TS and (d) SRS. Temperature response curves showing differences in the relationship between ecosystem respiration rates and temperature by ENSO phase during the wet season at (e) TS and (f) SRS, and during the dry season at (g) TS and (h) SRS. The shaded region highlights the wet season. At TS, there were 576 days (322 wet season; 254 dry season) in a La Niña phase, 304 days (188 wet season; 116 dry season) in an El Niño phase, 326 days (85 wet season; 241 dry season) in a post-La Niña phase, and 61 days (12 wet season; 49 dry season) in the post-El Niño phase, and 559 days (394 wet season; 165 dry season) in the neutral phase. At SRS, there were 576 days (308 wet season; 268 dry season) in a La Niña phase, 304 days (151 wet season; 153 dry season) in an El Niño phase, 326 days (85 wet season; 241 dry season) in a post-La Niña phase, 61 days (58 wet season; 3 dry season) in the post-El Niño phase, and 559 days in the neutral phase (401 wet season; 158 dry season).

Although TS ranged from a small CO_2_ sink to a small source on an annual basis over the 5 years, TS was often a source for CO_2_ during the wet season and a sink during the dry season (Table 2; Figs. 5 and 6), except in 2011 and 2013 when TS was a sink in both seasons. Changes in GEE relative to R_eco_ resulted in seasonal shifts in NEE, though there was no consistent pattern in dry season versus wet season response in GEE or R_eco_. The ratio of GEE to R_eco_ showed that in 2011 both seasons were comparable at TS and they differed the most in 2012 when a portion of the dry season was during a La Niña phase and the wet season was in a neutral phase (Table 2; S2a Fig.). CO_2_ uptake rates were generally higher in the dry season and during the exceptionally dry La Niña years at TS, which corresponded to drought conditions (Table 2; Fig. 5c). The mean annual dry season length at TS was 172 days for the 5 years. During years with La Niña phases, CO_2_ uptake was higher, dry seasons were 10 days longer on average, and TS was a greater sink for CO_2_ (Table 2). As a result of an extended drought in 2011 that occurred with 2 La Niña phases, TS was a sink for CO_2_ in both wet and dry seasons (Table 2).

Like patterns observed at TS, annual variation in NEE corresponded to changes in ENSO phase at SRS. Although SRS ranged from CO_2_ neutral to a small source of CO_2_ to the atmosphere annually and seasonally, CO_2_ release rates increased during seasons with La Niña phases. SRS was a small source of CO_2_ in most seasons, except in 2010 (El Niño) and the dry season of 2013 (neutral phase) (S2b Fig.).

Ecosystem respiration was the primary control on annual ecosystem carbon balance (Table 2) and dry season mean R_eco_ was often higher than wet season R_eco_, increasing CO_2_ release at SRS when the dry season was extended (S2b Fig.). Similar to TS, average daily CO_2_ uptake rates were higher in the dry season at SRS (Table 2). Even so, dry season mean daily R_eco_ rates were also higher than in the wet season (Table 2). Because R_eco_ increased relative to CO_2_ uptake in the dry season (Table 2; S2b Fig.), longer dry seasons were associated with greater CO_2_ source status at SRS. During the exceptionally wet year that corresponded to an El Niño phase (2010), GEE surpassed R_eco_ and the site became a larger sink for CO_2_ (Table 2). Although site differences were apparent in patterns of dry season CO_2_ exchange rates, there was no clear pattern in wet season CO_2_ uptake rates at TS and SRS (Table 2; S2Fig. a and b; S3 Fig. b and d). At TS wet season NEE during the neutral phase was most similar to that of the La Niña phase at TS (Table 2; S3 Fig. a and b), while at SRS all wet seasons were similar except the 2011 wet season which co-occurred with the strongest La Niña phase (Table 2; S3c Fig.).

Plots of dry season length versus dry season cumulative NEE (Fig. 4b and 4c) revealed differences by site. Although there were very few observations available since the study period included just 5 dry seasons, NEE at TS exhibited a negative linear relationship with dry season length (p = 0.0393; Fig. 4b), while NEE at SRS showed a positive linear relationship (p = 0.0206; Fig. 4c). These results demonstrate that the seasonal response in NEE rates differed between sites, and suggest that dry season length (and changes in dry season length) may control the CO_2_ source and sink status in the future. Although, the data set is not yet large enough to confirm the pattern between dry season length and dry season cumulative NEE as a characteristic of each site, this relationship is an important indication of how the sites respond differently to hydroperiods.

### Seasonal light and temperature response

In addition to site differences, ENSO phase and season altered photosynthetic capacity and ecosystem respiration (Table 3; Fig. 6). At both sites, photosynthetic capacity (*P_max_*) was greatest during La Niña and directly following La Niña phases compared to rates during neutral and El Niño phases, although at higher PAR values (>1500 µmol m^−2^ s^−1^) curves for La Niña and the neutral phase converged (Fig. 6a and 6b). Seasonal differences in light and temperature response curves were greater, and photosynthetic capacity and dark respiration (R_eco_) were consistently higher at TS, for all ENSO phases as compared to SRS (Table 3; Figure 6a and 6c). At both sites, the effect of El Niño and La Niña phase was also greater during the dry season. At SRS, photosynthetic capacity was similar for all ENSO phases during the dry season though the effect of El Niño and La Niña phases increased during the wet season (Table 3; Fig. 6b and 6d;). Overall, there was a small seasonal difference in photosynthetic capacity, which was higher on average in the wet season than in the dry season at SRS. At both sites, differences in photosynthetic capacity by ENSO phase were greatest at higher PAR values (>1000 µmol m^−2^ s^−1^).

**Table 3:** Model estimates from Eq.6 and 7 for TS and SRS by ENSO phase and season.

			Light Response Curves		Temperature Response Curves		
Site	ENSO	Season	*α*	*P_max_*	*R_eco_*	*R*_0_	*b*
TS	El Niño	Dry	−0.0077	−4.28	1.2063	0.4361	0.0482
	La Niña	Dry	−0.0088	−5.7676	1.4031	0.6134	0.0335
	Post-El Niño	Dry	−0.02	−5.4761	1.8287	1.0559	0.0058
	Post-La Niña	Dry	−0.0185	−6.0613	1.9068	0.7596	0.017
	Neutral	Dry	−0.00643	−4.8674	1.2702	0.9797	0.00598
	El Niño	Wet	−0.0122	−1.9836	0.8323	0.4096	0.0319
	La Niña	Wet	−0.0134	−3.4053	1.147	0.635	0.0183
	Post-La Niña	Wet	−0.0209	−3.6654	1.2564	0.209	0.0608
	Neutral	Wet	−0.00466	−3.7001	0.7102	0.4088	0.0238
SRS	El Niño	Dry	−0.0149	−2.0916	0.8782	0.3408	0.0344
	La Niña	Dry	−0.0138	−2.3326	0.9367	0.3846	0.0414
	Post-El Niño	Dry	−0.0034	−1.7188	0.254		
	Post-La Niña	Dry	−0.0643	−4.1691	2.9143	0.7255	0.0198
	Neutral	Dry	−0.00972	−2.1901	0.7655	0.62	0.00399
	El Niño	Wet	−0.0281	−2.2678	1.5688	0.2496	0.0539
	La Niña	Wet	−0.0217	−2.5837	1.1998	0.3184	0.0409
	Post-La Niña	Wet	−0.0359	−3.1999	1.6757	0.3089	0.0418
	Neutral	Wet	−0.00693	−2.2765	0.8207	0.3034	0.0267

The relationship between temperature and R_eco_ differed between ENSO phases, and R_eco_ showed distinct seasonal patterns in temperature sensitivity (Table 3; Fig. 6e to 6h) at TS and SRS. During the wet season at both sites, R_eco_ was less sensitive to temperature changes. At TS temperature effects associated with ENSO phases were greater at lower temperatures (Table 3; Fig. 6e and 6f). At higher temperatures R_eco_ was more sensitive to changes in temperature during all phases at both sites, a response that was enhanced during the dry season (Table 3; Fig. 6g and 6h). At SRS, the differences among ENSO phases were small except at high temperatures (>24°C) during the wet season, while at TS the differences among ENSO phases were consistently large at lower temperatures and converged at high temperatures (Fig. 6e to 6h). Similar to patterns observed in light response curves, respiration rates were higher at TS than at SRS, and temperature patterns associated with R_eco_ also showed greater release of CO_2_ at both sites in the dry season versus the wet season (Fig. 6e to 6h).

### The effect of ENSO phase, precipitation, and season on daily CO_2_ exchange rates and water level

After pre-whitening, some small (<0.05) but statistically significant autocorrelation remained in pre-whitened series; however, this sensitivity resulted from the large number of observations available and was judged to be biologically insignificant [47]. Differencing was required for water level and PDSI time series due to the lack of stationarity at both sites. Non-stationarity indicates a lack of stability in the mean of these variables over time, further suggesting that there were significant changes in hydroperiods at both sites. By including differenced variables in models we evaluated how changes in water level and PDSI influenced NEE, R_eco_, and GEE. In response to evidence of 1-month lagged teleconnections for ENSO phase effects on precipitation and average daily maximum and minimum temperatures (Table 1), lagged El Niño and La Niña phase indicators were included in time series models of CO_2_ exchange rates.

Models for NEE (S4a and S5a Figs.), had a significant lagged 1-day MA [MA(1)] component, as well as significant AR components at a lag of 1-, and 2-days (p<0.0001;Table 4). At TS, Δwater level (p = 0.01) and the quantity of rain (mm day^−1^; p<0.0001) had significant positive relationships with NEE, showing that as the change in water level and the quantity of daily rainfall increased, net CO_2_ uptake decreased (higher NEE; Table 4). Post-La Niña phases in the dry season were associated with significantly lower NEE (higher net CO_2_ uptake) compared to neutral and El Niño phases at TS (p = 0.0039); however, post-La Niña phases in the dry season at SRS were associated with greater NEE (lower net CO_2_ uptake; p = 0.0394). Moreover, there was a significant increase in NEE at TS (higher net CO_2_ uptake) the day after rain. At TS where hydroperiods were shorter, the effect of rain and post-La Niña phase during the dry season were significantly stronger than at SRS (Table 4). The quantity of rain was the strongest driver of NEE at both sites.

**Table 4:** Parameter estimates from ARIMA models of daily NEE by site.

	Taylor Slough					Shark River Slough			
Parameter	Estimate	Standard Error	t Value	Approx		Estimate	Standard Error	t Value	Approx
				Pr>|t|					Pr>|t|
MA(1)	0.7760	0.0304	25.52	<.0001		0.7652	0.0324	23.66	<.0001
AR(1)	1.2808	0.0418	30.67	<.0001		1.2954	0.0435	29.77	<.0001
AR(2)	−0.2998	0.0385	−7.78	<.0001		−0.3153	0.0401	−7.86	<.0001
Day After Rain	0.0346	0.0128	2.70	0.007		0.0171	0.0109	1.56	0.118
Rain (mm)	0.0073	0.0005	14.23	<.0001	*	0.0027	0.0004	6.21	<.0001
ΔWater Level (m)	0.3464	0.1415	2.45	0.0144		0.1377	0.1997	0.69	0.4904
Post-La Niña (Wet Season)	−0.4943	0.1333	−3.71	0.0002		−0.0755	0.0954	−0.79	0.4292
Post-La Niña (Dry Season)	−0.2187	0.0758	−2.89	0.0039	*	0.1395	0.0678	2.06	0.0394

MA(1) is the estimated moving average term at a 1-period lag (1 day), and AR (1) and AR(2) are the estimated autoregressive terms at a 1- and 2-period lags (1 and 2 days). Lagged values of independent variables are denoted similarly. Asterisks denote significant differences between sites. *Day after rain* is an indicator for the first rain free day, *Rain* is the quantity of precipitation (mm), *ΔWater Level* is the change in water level from one day to the next, *Post-La Niña (Wet Season)* is an indicator for the time directly following a La Niña phase in the wet season, and *Post-La Niña (Dry Season)* is an indicator for the time directly following a La Niña phase in the dry season.

Models for R_eco_ (S4b and S5b Figs.), at both sites had a significant 1-day MA [MA(1)], as well as significant AR components at a lag of 1- and 2-days (p<0.001; Table 5). The day after rain (p = 0.0047), and post-La Niña phases in the dry season (p = 0.0291) reduced daily ecosystem respiration rates at TS (Table 5), while the quantity of rain (p = 0.0596), and post-El Niño phases in the dry season were associated with an increase in R_eco_. At SRS, post-La Niña phases in the dry season (p = 0.0002) were significantly linked to increased R_eco_ (Table 5), and the effect of post-La Niña phases in the dry season was larger at SRS where it was associated with increased R_eco_. At SRS, post-La Niña phases had the greatest impact on R_eco_, while at TS R_eco_ had the strongest association with the day following rain events.

**Table 5:** Parameter estimates from ARIMA models of daily R_eco_ by site.

	Taylor Slough					Shark River Slough			
Parameter	Estimate	Standard Error	t Value	Approx		Estimate	Standard Error	t Value	Approx
				Pr>|t|					Pr>|t|
MA(1)	0.8786	0.0211	41.6	<.0001		0.8209	0.0226	36.29	<.0001
AR(1)	1.5960	0.0355	44.95	<.0001	*	1.3410	0.0372	36.09	<.0001
AR(2)	−0.5962	0.0355	−16.82	<.0001	*	−0.3415	0.0371	−9.21	<.0001
Day After Rain	−0.0217	0.0077	−2.83	0.0047		0.0045	0.0089	0.51	0.6126
Rain (mm)	0.0005	0.0003	1.88	0.0596		−0.0002	0.0004	−0.68	0.4943
Post-La Niña (Dry Season)	−0.0908	0.0416	−2.18	0.0291	*	0.1544	0.0417	3.7	0.0002
Post-El Niño (Dry Season)	0.1479	0.0769	1.92	0.0545		0.0017	0.0757	0.02	0.982

MA(1) is the estimated moving average term at a 1- period lag (1 day), and AR (1) and AR(2) are the estimated autoregressive terms at a 1- and 2- period lags (1 and 2 days). Lagged values of independent variables are denoted similarly. Asterisks denote significant differences between sites. *Day after rain* is an indicator for the first rain free day, *Rain* is the quantity of precipitation (mm), *Post-La Niña (Dry Season)* is an indicator for the time directly following a La Niña phase in the dry season, and *Post-El Niño (Dry Season)* is an indicator for the time directly following a La Niña phase in the dry season.

Similar to NEE, models for GEE had a significant 1-day lagged MA [MA(1)] and AR [AR(1)] component at both sites (p<0.0001;Table 6; S4c and S5c Figs.). The day of rain (p<0.0001), day after rain (p = 0.0015), quantity of rain (p<0.0001), and Δwater level (p<0.0001) had significant positive relationships with GEE at TS (Table 6). At SRS, days with precipitation (p<0.0001) and Δwater level (p = 0.005) had positive relationships with GEE (Table 6). Rain had a stronger effect at TS than at SRS, and rain had the strongest effect on GEE at SRS, while Δwater level had the strongest effect on GEE at TS (Table 6).

**Table 6:** Parameter estimates from ARIMA models of daily GEE by site.

	Taylor Slough					Shark River Slough			
Parameter	Estimate	Standard Error	t Value	Approx		Estimate	Standard Error	t Value	Approx
				Pr>|t|					Pr>|t|
MA(1)	0.6113	0.0213	28.67	<.0001		0.6406	0.0205	31.21	<.0001
AR(1)	0.9975	0.0017	602.66	<.0001		0.9967	0.0020	493.35	<.0001
Rain Day	0.1263	0.0145	8.7	<.0001	*	0.0609	0.0135	4.52	<.0001
Day After Rain	0.0484	0.0152	3.18	0.0015		−0.0113	0.0140	−0.81	0.4207
Rain (mm)	−0.0035	0.0007	−5.12	<.0001		−0.0008	0.0007	−1.11	0.2651
ΔWater Level (m)	1.8698	0.2132	8.77	<.0001		1.4030	0.4997	2.81	0.005
Post-La Niña (Wet Season)	−0.2051	0.1337	−1.53	0.125		0.0430	0.0843	0.51	0.6098

MA(1) is the estimated moving average term at a 1-period lag (1 day), and AR (1) is the estimated autoregressive term at a 1-period lag (1 day). Lagged values of independent variables are denoted similarly. Asterisks denote significant differences between sites. *Rain Day* is an indicator for days with precipitation>0, *Day After Rain* is the first rain-free day, *Rain* is the quantity of precipitation (mm), *ΔWater Level* is the change in water level from one day to the next, and *Post-La Niña (Wet Season)* is an indicator for the time directly following a La Niña phase in the wet season.

Models for the water index had a significant 1-day lagged MA [MA(1)] and AR [AR(1)] component at both sites (p<0.0001; Table 7; S4d and S5d Figs.). At TS, La Niña phases during the wet season (p<0.0001) and post-La Niña phases (wet season; p<0.0001) were associated with lower than average water levels compared to neutral and El Niño phases (Table 7). Water levels at TS were higher than the seasonal average the day of rain (0.0545), the day after rain (p = 0.162), during dry season EL Niño phases (p<0.0001), and throughout post-La Niña phases that occurred at the end of the dry season (p<0.0001). At SRS, the day of rain (p = 0.0349), the day after rain (p = 0.0066) and post-La Niña phase at the end of the dry season (p<0.0001) were associated with higher than average water levels (Table 7). La Niña and post-La Niña phases (wet season; p<0.0001) were associated with lower than average water levels. The effect of La Niña (wet season) and El Niño (dry season) phases were significantly greater at TS than at SRS, and La Niña and post- La Niña phases were the strongest predictors of the water index (Table 7).

**Table 7:** Parameter estimates from ARIMA models of the daily water index by site.

	Taylor Slough					Shark River Slough			
Parameter	Estimate	Standard Error	t Value	Approx		Estimate	Standard Error	t Value	Approx
				Pr>|t|					Pr>|t|
MA(1)	−0.2101	0.0262	−8.02	<.0001		−0.1826	0.0262	−6.97	<.0001
AR(1)	0.9924	0.0031	318.42	<.0001		0.9882	0.0040	246.96	<.0001
Rain Day	0.0128	0.0066	1.92	0.0545		0.0109	0.0052	2.11	0.0349
Day After Rain	0.0097	0.0069	1.4	0.162		0.0145	0.0053	2.72	0.0066
Rain (mm)	0.0024	0.0002	10.54	<.0001	*	0.0006	0.0002	3.52	0.0004
La Niña (Wet Season)	−1.6858	0.0608	−27.71	<.0001	*	−0.8015	0.0541	−14.83	<.0001
El Niño (Dry Season)	0.9664	0.0673	14.36	<.0001	*	−0.0793	0.0790	−1	0.3156
Post-La Niña (Wet Season)	−1.6847	0.0843	−19.99	<.0001	*	−1.2802	0.0535	−23.92	<.0001
Post-La Niña (Dry Season)	0.6185	0.0487	12.69	<.0001	*	0.3928	0.0390	10.07	<.0001
Post-El Niño (Dry Season)	−0.3547	0.0989	−3.59	0.0003		−0.4239	0.0851	−4.98	<.0001

MA(1) is the estimated moving average term at a 1-period lag (1 day), and AR (1) is the estimated autoregressive term at a 1-period lag (1 day). Lagged values of independent variables are denoted similarly. Asterisks denote significant differences between sites. *Rain Day* is an indicator for days with precipitation>0, *Day After Rain* is the first rain free day, *Rain* is the quantity of precipitation (mm), *La Niña (Wet Season)* is an indicator for wet season La Niña phases, *El Niño (Dry Season)* is an indicator for dry season El Niño phases, *Post-La Niña (Wet Season)* is an indicator for the time directly following a La Niña phase in the wet season, *Post-La Niña (Dry Season)* is an indicator for the time directly following a La Niña phase in the dry season, and *Post-El Niño (Dry Season)* is an indicator for the time directly following a El Niño phase in the dry season.

## Discussion

*The goal of this research was to understand the relationship between ENSO phases and CO_2_ exchange rates (NEE, R_eco_ and GEE) in Everglades freshwater marsh ecosystems.* The relationships between ENSO extremes, precipitation and hydrology in the Everglades region suggest that El Niño and La Niña phases could be important for C dynamics [1], [3], [48], [49], [50], [51], [52], [53], [54], [55]. The results presented here show that climate teleconnections have significant controls on Everglades CO_2_ dynamics, demonstrating that in addition to climate change and water management, ENSO is an additional source of variation in C cycling in Everglades freshwater ecosystems.

### Annual fluctuations in season length and intensity

Interannual variability in precipitation can be large in the Everglades region [21], [56], which was the case throughout the study period. Reduced precipitation prior to the onset of the wet season resulted in shorter wet seasons and lower water levels, though annual precipitation was unchanged. Cumulative precipitation in January through March was related to wet season length (Fig. 4a). Although more data is needed to validate this relationship, previous research suggests dry season rainfall from October to April largely determines season intensity [3], [56]. Higher precipitation from January to March was also associated with longer and wetter than average wet seasons. Interannual variation in the onset and length of seasons can have a significant effect on the magnitude of ecosystem primary production [57], and changes in season intensity can either suppress or enhance production [58]. Results suggest that in Everglades ecosystems dry season length has a strong relationship with annual NEE (S6 Fig.). Knowing that precipitation patterns were driving the variations observed in season length and intensity, we examined the co-occurrence of ENSO phases, previously found to alter season intensity in the Everglades region [3], [55], to PDSI defined season intensity.

Abnormal precipitation and water level patterns over the study period coincided with El Niño and La Niña phases. Changes in ENSO phase have been associated with shifts in the position of the midlatitude jet, which is important for patterns in frontal precipitation. In the Everglades where frontal precipitation is the main source of dry season rainfall [20], changes in ENSO phase can have a significant effect on hydroperiods. During El Niño (La Niña) phases, the equatorial (poleward) displacement of the midlatitude jet increases (decreases) frontal precipitation in the southeastern United States [1], [48], [49]. Studies have shown that in Florida, El Niño is positively correlated with winter (dry season) precipitation, explaining up to 34% of dry season precipitation variability [4]. Here, El Niño and La Niña phases have been shown to reduce seasonal differences in rainfall without altering annual precipitation inputs [55].

Results found by Beckage et al. [3] and our study show that El Niño (La Niña) phases were correlated with increased (decreased) rainfall and water levels in the Everglades region. Time series analysis of the water index at TS and SRS reinforced the relationships previously found between precipitation patterns and ENSO phase. At both TS and SRS, La Niña and post- La Niña phases were associated with lower than average water levels and at TS water levels were higher than the seasonal average during the El Niño phase (dry season). The effect of La Niña (wet season) and El Niño (dry season) phases were significantly greater at TS than at SRS, and La Niña and post- La Niña phases were the strongest predictors of the water index at both sites. These results support the previously observed patterns in ENSO phases [3], precipitation, and hydrology and show that El Niño and La Niña phases induced fluctuations in season intensity (+ and -, respectively) compared to neutral phases, which affect season length, and have important implications for annual CO_2_ exchange rates.

### Seasonal patterns in CO_2_ exchange rates

Hydroperiods have shaped soil conditions and species composition at each site in ways that have led to different seasonal patterns in CO_2_ exchange rates. Hydroperiods alter ecosystem production by interfering with exposed leaf area [11], [12], [16], triggering senescence (S. Oberbauer, unpublished data), and allowing CO_2_ fixation within the water column [16]. Seasonal changes in photosynthetic capacity (Fig. 6) and the relationship between respiration rates and temperature support the patterns previously found in Everglade freshwater marsh studies [11], [12]. Knowing that season intensity changed with El Niño (+) and La Niña (-) phases (Fig. 3a), we expected and saw a magnification of the site-specific seasonal response in CO_2_ exchange rates during and directly following El Niño and La Niña phases. The lag in El Niño and La Niña phase effect on CO_2_ exchange rates is the result of the effect of extreme ENSO phases on water levels during transition periods. El Niño (La Niña) phases increase (decrease) surface water levels during seasonal transitions and, major drainages often contain no water during transitions in a La Niña phase [3]. Results suggest that Everglades freshwater marsh ecosystems are more similar during El Niño and neutral phases when water levels are higher. This is further reinforced by time series analysis, which detected significant changes in CO_2_ exchange rates between ENSO and post-ENSO phases at TS and SRS.

The sites differed in their response to ENSO phases. At TS, CO_2_ exchange rates were sensitive to El Niño and La Niña phase in both the wet and dry seasons, while at SRS the effect of El Niño and La Niña phase on the wet season was not as strong as the effect in the dry season. La Niña phases resulted in higher photosynthetic capacity and greater seasonal net carbon uptake rates in both seasons at TS compared to neutral and El Niño phases. While La Niña increased photosynthetic capacity at SRS, the increase in R_eco_ reduced net exchange rates compared to neutral and El Niño phases. As a result of changes in GEE relative to R_eco_, El Niño led to greater net CO_2_ uptake at SRS during the dry season compared to La Niña and neutral phases. As water levels and precipitation increased, NEE and GEE also increased at both sites (less CO_2_ and net CO_2_ uptake). At TS where hydroperiods were shorter, the effect of rain and the post-La Niña phase during the dry season were significantly stronger than at SRS (Table 4). Rain was one of the strongest drivers of CO_2_ dynamics and these results indicate that there is a significant relationship between El Niño and La Niña phase and season intensity, either creating conditions wetter (+) or dryer (-) than normal, which magnifies CO_2_ exchange rates at TS and SRS.

### Annual patterns in CO_2_ exchange rates

The results presented here show that the length and intensity of the wet and dry season varied annually with climate patterns. Considering the site-specific response to season, these results support our hypothesis that variations in season length would explain interannual fluctuations in NEE (S6 Fig.), R_eco_, and GEE. At TS where mean GEE surpassed R_eco_ during the dry season, an increase in dry season length and intensity amplified the site's net CO_2_ uptake rates. Annually, SRS was usually a small source of CO_2_, although when wet season conditions intensified during El Niño, net CO_2_ uptake increased. The effect of ENSO phase also differed by site, showing that longer hydroperiods mute the effect of climate fluctuations on CO_2_ exchange rates, and as water levels decline the system becomes more vulnerable to climate. The ecosystem's sensitivity to climate fluctuations has important implications for water management and climate change [59]. The uncertainty of climate change makes it important to understand how ecosystems respond to climate events and how these responses aggregate to form trends in net CO_2_ exchange rates.

### Effect of climate change and water management on net CO_2_ exchange rates

In sub-tropical ecosystems, phenology is less sensitive to temperature and photoperiod, and more tuned to seasonal shifts in precipitation [60], [61], [62]. Such shifts are expected to occur in concert with rising global temperatures, but both the direction and magnitude of change vary regionally [58], [63]. As climate change has the potential to alter hydrologic regimes, we can expect to see greater variations in CO_2_ exchange rates. Shifts in water management and land use change (e.g., conversion to agriculture and urban development) could also significantly alter both hydrology and CO_2_ dynamics in the Everglades region, making it important to develop a baseline understanding of how hydroperiod drives changes in CO_2_ dynamics and how climate alters hydrology. With water managers striving to adjust hydroperiods closer to natural values, in the future we might expect water levels in TS to increase, offsetting changes in climate by maintaining current patterns in hydrology. Alternatively, we might anticipate higher water levels to increase hydroperiods, making the system less sensitive to climate change altogether. With longer hydroperiods, SRS will likely remain less sensitive to changes in climate and land management. As a result, the two sites will likely behave more similarly in the future as SRS remains neutral or a very small source of CO_2_ to the atmosphere and TS becomes more neutral.

Patterns observed in ENSO phases and the co-occurrence of extreme wet and dry seasons suggest changes in climate patterns can significantly alter ecosystem function. Equatorial Pacific SST during the past half century show a clear warming trend that is consistent with global warming [64], and El Niño and La Niña phases are expected to continue increasing in severity and frequency [3], [17], [58]. Moreover, as a result of warming SST, ENSO amplitude may become even stronger, intensifying feedbacks relevant to ENSO phases [64]. If the frequency and intensity of strong climatic disturbances increases beyond historical averages, altered disturbance regimes have the capacity to significantly modify ecosystem processes [65]. ENSO phases have been linked to climate anomalies [53] and CO_2_ dynamics [66] on a global scale [53], making it crucial to analyze the importance of ENSO extremes and other cyclic climatic phenomenon on the variability of terrestrial carbon cycling.

### Study Limitations

This research shows that the length and intensity of the wet and dry season vary annually with ENSO phase in the subtropical Everglades region where dry season precipitation is dependent on frontal systems. Changes in season length and intensity are also correlated with CO_2_ exchange rates showing that extreme ENSO phases magnify the site-specific seasonal response in freshwater marsh ecosystems. Although a longer time series is required to verify that this relationship is persistent, this research provides initial insights into an important driver of seasonal and inter-annual variation in CO_2_ exchange rates in Everglades ecosystems.

## Supporting Information

S1 Fig **ARIMA model versus observed data of (a) precipitation, (b) maximum temperature and minimum temperature.** Long-term weather data were obtained from NCDC Royal Palm Ranger Station (25°23′N/80°36′W), where NOAA surface meteorological data was available from 1964 to 2013.
(TIF)

S2 Fig **The ratio of GEE to R_eco_ at TS and SRS.** Seasonal patterns in the ratio of GEE to R_eco_ at (a) TS and (b) SRS shows that there is no clear pattern in wet season CO_2_ uptake rates and the (c) annual ratio of GEE to R_eco_ were most similar during El Niño and neutral phases at TS and SRS.
(TIF)

S3 Fig **Seasonal NEE at TS and SRS.** At TS patterns in (a) wet and (b) dry season NEE shows that net CO_2_ uptake was greatest in years associated with La Niña phases (2011 and 2012). At SRS (a) wet and (b) dry season NEE suggests that the greatest net CO_2_ uptake occurred in years associated with El Niño (2010) and neutral phases (2013).
(TIF)

S4 Fig **ARIMA model versus observed data of (a) NEE, (b) R_eco_, (c) GEE, and (d) the water index for TS.** An intervention time series approach was used to identify and model the relationship between CO_2_ dynamics (*NEE, GEE*, and *R_eco_*) and a set of explanatory variables over a 5-year time series of daily data (2009 to 2013).
(TIF)

S5 Fig **ARIMA model versus observed data of (a) NEE, (b) R_eco_, (c) GEE, and (d) the water index for SRS.** An intervention time series approach was used to identify and model the relationship between CO_2_ dynamics (*NEE, GEE*, and *R_eco_*) and a set of explanatory variables over a 5-year time series of daily data (2009 to 2013).
(TIF)

S6 Fig **The relationship between season length and NEE at TS and SRS.** NEE had a negative relationship with dry season length at (a) TS and a positive relationship with dry season length at (b) SRS. Annual NEE was positively correlated with wet season length at (c) TS and negatively correlated with wet season length at (d) SRS.
(TIF)
