# Supplementary figures and images for: El Niño Southern Oscillation (ENSO) Enhances CO2 Exchange Rates in Freshwater Marsh Ecosystems in the Florida Everglades
Source: PLoS One. 2014 Dec 18;9(12):e115058. doi: 10.1371/journal.pone.0115058 (PMC4270789; doi:10.1371/journal.pone.0115058)

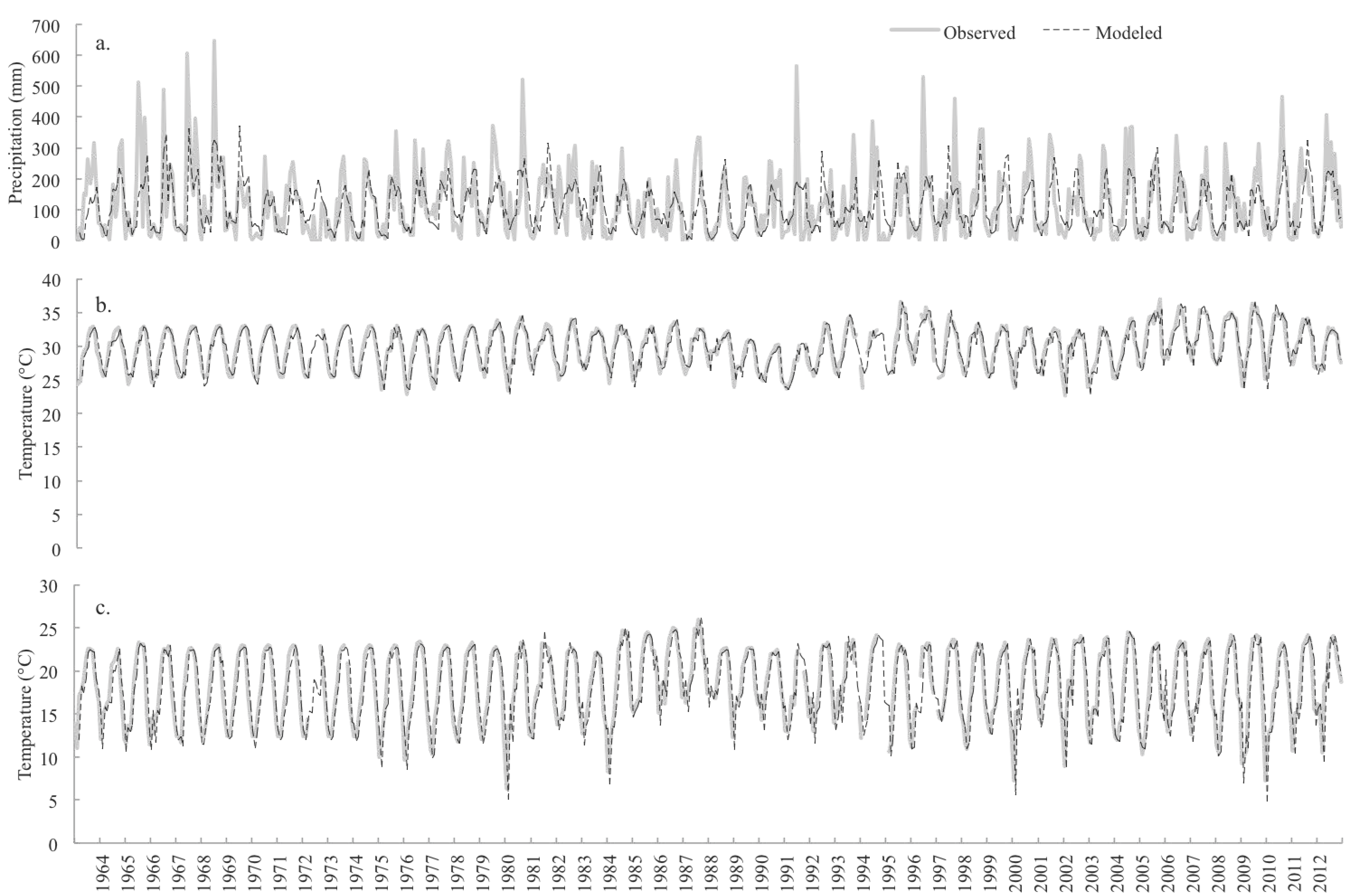

Supplement: S1 Fig — ARIMA model versus observed data of (a) precipitation, (b) maximum temperature and minimum temperature. Long-term weather data were obtained from NCDC Royal Palm Ranger Station (25°23′N/80°36′W), where NOAA surface meteorological data was available from 1964 to 2013. (TIF) [file pone.0115058.s001.tif]

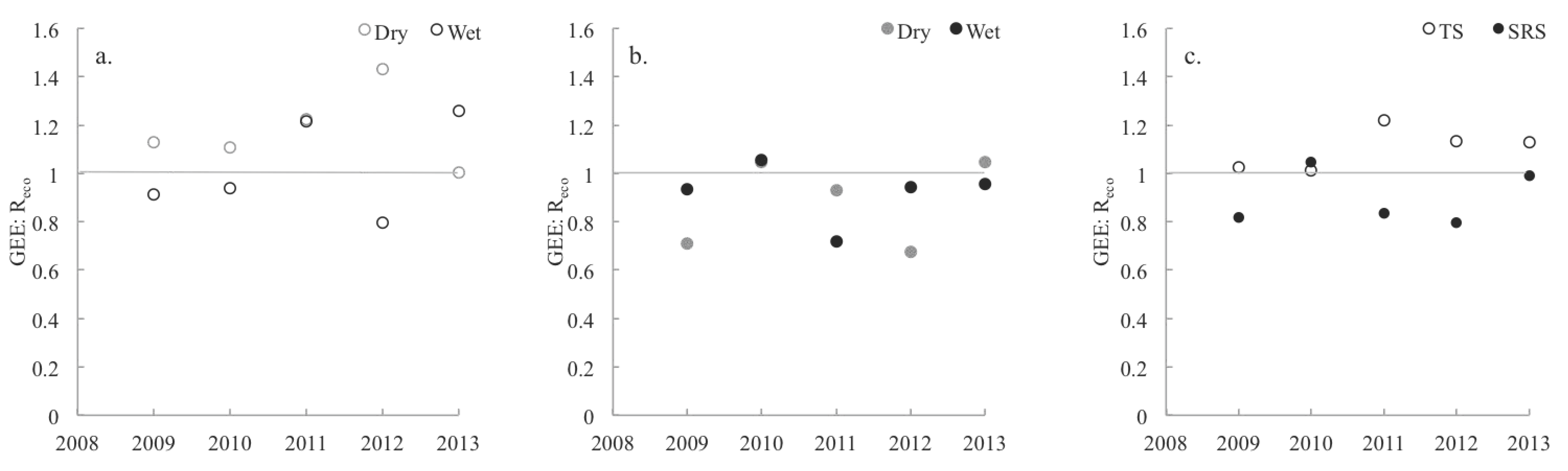

Supplement: S2 Fig — The ratio of GEE to Reco at TS and SRS. Seasonal patterns in the ratio of GEE to Reco at (a) TS and (b) SRS shows that there is no clear pattern in wet season CO2 uptake rates and the (c) annual ratio of GEE to Reco were most similar during El Niño and neutral phases at TS and SRS. (TIF) [file pone.0115058.s002.tif]

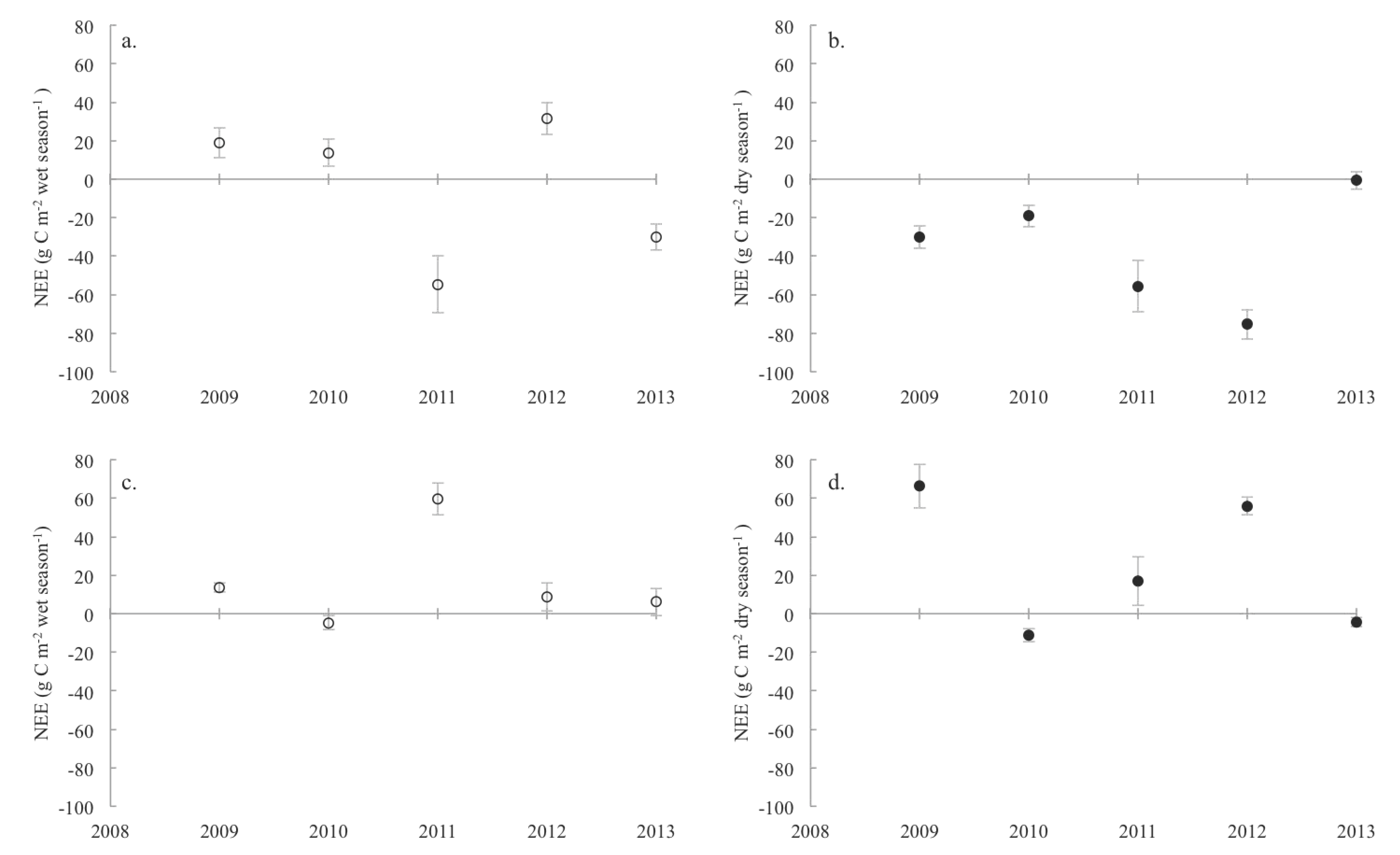

Supplement: S3 Fig — Seasonal NEE at TS and SRS. At TS patterns in (a) wet and (b) dry season NEE shows that net CO2 uptake was greatest in years associated with La Niña phases (2011 and 2012). At SRS (a) wet and (b) dry season NEE suggests that the greatest net CO2 uptake occurred in years associated with El Niño (2010) and neutral phases (2013). (TIF) [file pone.0115058.s003.tif]

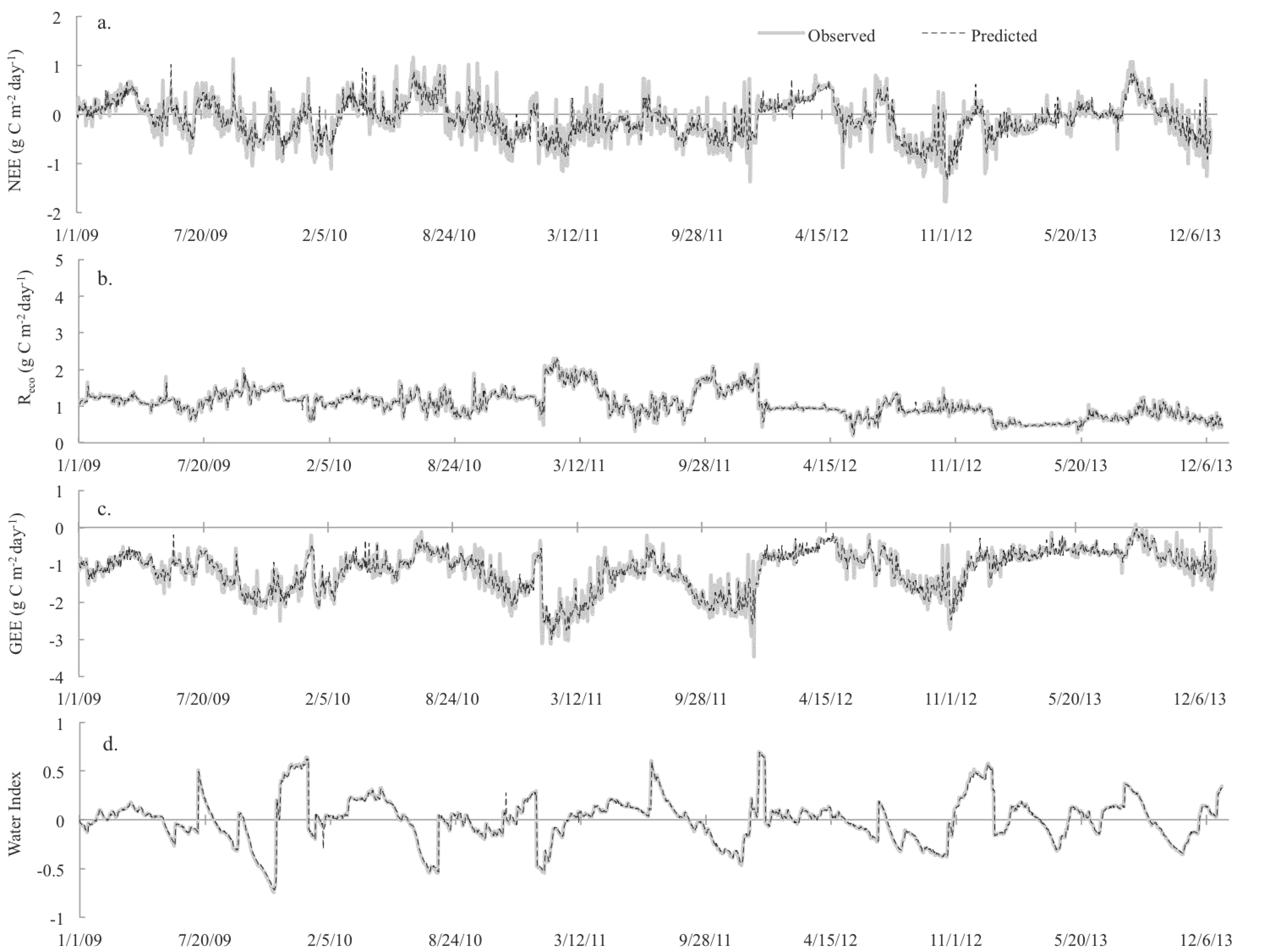

Supplement: S4 Fig — ARIMA model versus observed data of (a) NEE, (b) Reco, (c) GEE, and (d) the water index for TS. An intervention time series approach was used to identify and model the relationship between CO2 dynamics (NEE, GEE, and Reco) and a set of explanatory variables over a 5-year time series of daily data (2009 to 2013). (TIF) [file pone.0115058.s004.tif]

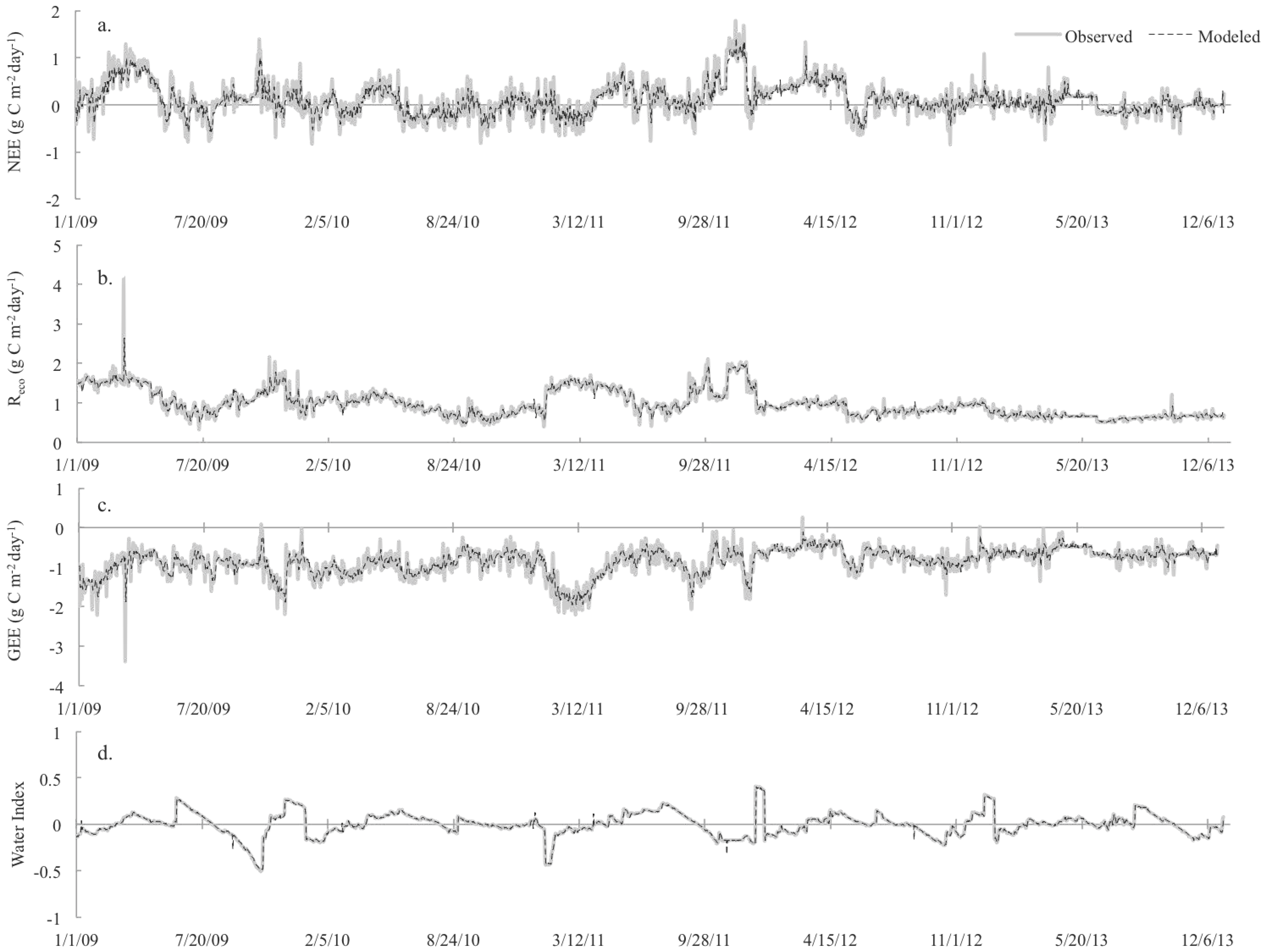

Supplement: S5 Fig — ARIMA model versus observed data of (a) NEE, (b) Reco, (c) GEE, and (d) the water index for SRS. An intervention time series approach was used to identify and model the relationship between CO2 dynamics (NEE, GEE, and Reco) and a set of explanatory variables over a 5-year time series of daily data (2009 to 2013). (TIF) [file pone.0115058.s005.tif]

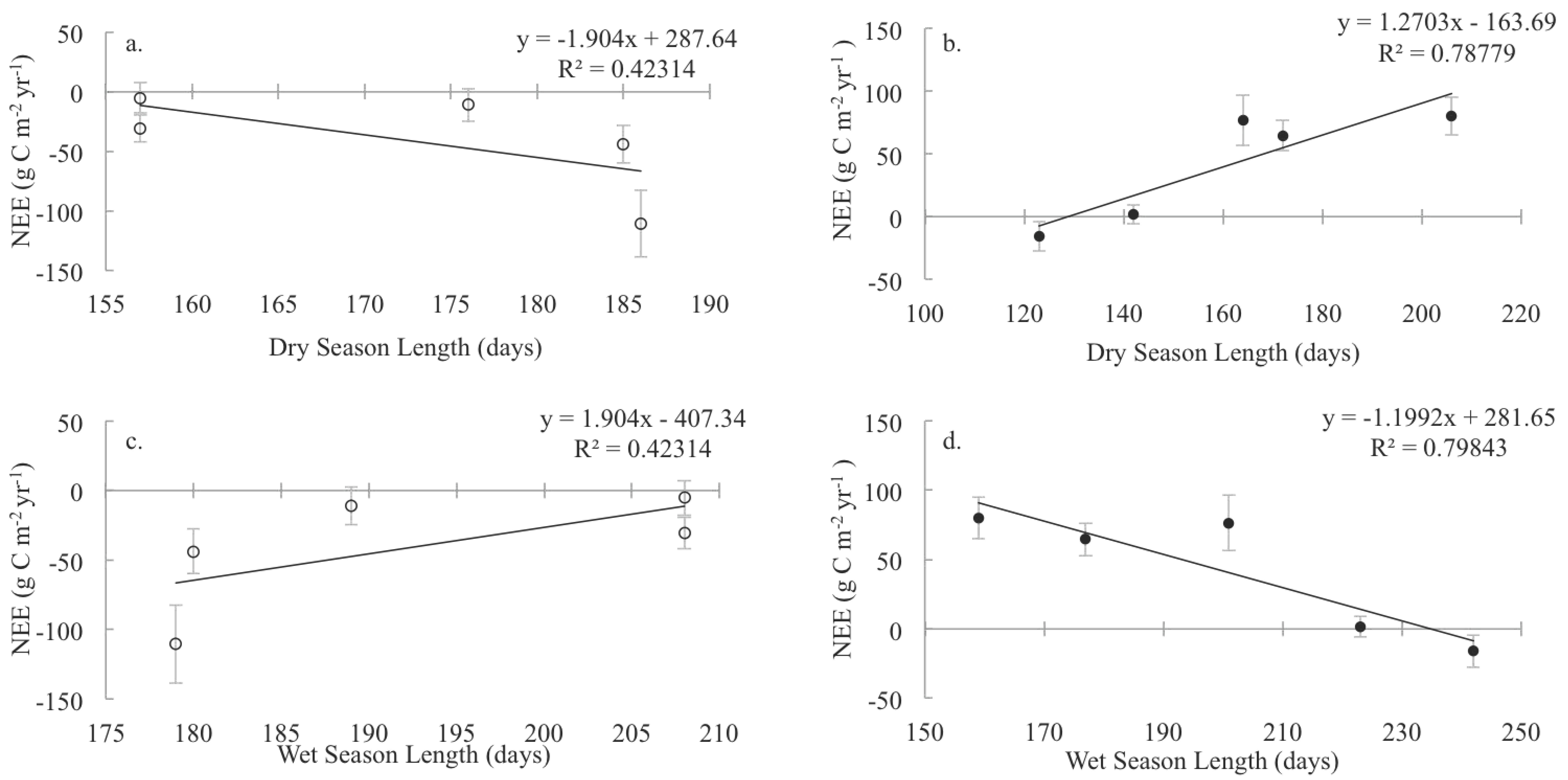

Supplement: S6 Fig — The relationship between season length and NEE at TS and SRS. NEE had a negative relationship with dry season length at (a) TS and a positive relationship with dry season length at (b) SRS. Annual NEE was positively correlated with wet season length at (c) TS and negatively correlated with wet season length at (d) SRS. (TIF) [file pone.0115058.s006.tif]
